# rs6837671A>G in *FAM13A* Is a Trans-Ethnic Genetic Variant Interacting with Vitamin D Levels to Affect Chronic Obstructive Pulmonary Disease

**DOI:** 10.3390/jpm11020084

**Published:** 2021-01-30

**Authors:** Said El Shamieh, Ali Salami, Mirna Fawaz, Rania Jounblat, Mirna Waked, Rajaa Fakhoury

**Affiliations:** 1 Department of Medical Laboratory Technology, Faculty of Health Sciences, Beirut Arab University, Beirut P.O. Box 115020, Lebanon; rfakhoury@bau.edu.lb; 2 Rammal Hassan Rammal Research Laboratory, PhyToxE Research Group, Faculty of Sciences, Lebanese University, Nabatieh P.O. Box 6573/14, Lebanon; a.salami@ul.edu.lb; 3 Department of Nursing, Faculty of Health Sciences, Beirut Arab University, Beirut P.O. Box 115020, Lebanon; mirna.fawaz@bau.edu.lb; 4 Department of Life and Earth Sciences, Faculty of Sciences II, Lebanese University, Fanar P.O. Box 26110217, Lebanon; rjounblat@ul.edu.lb; 5 Department of Pulmonology, St George Hospital University Medical Center, Achrafieh Beirut, P.O. Box 166 378, Lebanon; mirnawaked1@gmail.com; 6 Faculty of Medicine, University of Balamand, Achrafieh Beirut, P.O. Box 166 378, Lebanon

**Keywords:** chronic obstructive pulmonary disease, single nucleotide polymorphism, vitamin D, interaction, meta-analysis

## Abstract

(1) Background and objectives: Chronic obstructive pulmonary disease (COPD) is a leading cause of mortality throughout the world. In addition to genetics, increasing evidence suggests that Vitamin D (VitD) might be involved in different pathogenic mechanisms in COPD. Furthermore, the prevalence of VitD insufficiency is exceptionally high in COPD patients and increases with the severity. Based on the above, we first tested the relation between the top 10 single nucleotide polymorphisms from genome-wide association studies and the risk of COPD. Then, we investigated whether VitD levels might also have a role in COPD. A meta-analysis followed, combining our participants with previously published European and non-European populations (15,716 cases and 48,107 controls). (2) Methods: 631 Lebanese participants were recruited, of which ~28% were affected with COPD. Demographic and clinical data were collected, and DNA was genotyped using Kompetitive allele-specific PCR (KASPT^M^). Adjusted multiple logistic regression models were used. Bonferroni corrections were also applied. The statistical power was also assessed. (3) Results: Both rs6837671A>G in *FAM13A* and VitD levels were significantly associated with increased risk of COPD (OR = 1.75, *p* = 0.01, and OR = 3.10, *p* < 0.001 respectively). An interaction between rs6837671A>G in *FAM13A* and VitD levels, which increased COPD risk, was found (OR = 3.35 and *p* < 0.001). The meta-analysis showed that rs6837671G increases COPD risk in populations from different origins; Europeans, Asians, and now in Middle-Eastern. (4) Conclusions: Our results suggest that rs6837671A>G in *FAM13A* is a trans-ethnic genetic variant that interact with VitD to affect COPD.

## 1. Introduction

Chronic obstructive pulmonary disease (COPD) is a leading cause of mortality worldwide [1]. According to the world health organization, more than 90% of COPD deaths occur in low­ and middle­ income countries [2]. In Lebanon, COPD’s prevalence is 9.7% from the general population aged 40 years and over [3]. COPD is characterized by persistent and progressive airflow limitation diagnosed by lung function testing [1]. This disease is a complex trait having both environmental and genetic risk factors [4]. Although cigarette smoking is the most significant environmental, not all smokers develop COPD, and lung function decline among smokers varies significantly [5], which suggests that additional factors, including genetics, play an essential role in the susceptibility to COPD [6]. COPD’s genetic etiology includes common and rare variants having small and large effect sizes, respectively [6]. 

The role of vitamin D (VitD) in calcium and bone homeostasis is well documented [7]. In the decade, it has been recognized that in addition to this classical function, VitD is implicated in various processes, including immunity, inflammation, and pulmonary biology [8]. The deficiency in VitD levels is prevalent in patients with COPD [9]. Specifically, it is present in 40–80 % of the affected individuals and correlated with the disease severity [9]. The presence of VitD receptors on immunity cells [10] and the high prevalence of VitD deficiency among COPD patients have given rise to the hypothesis that VitD might be implicated in COPD’s pathogenesis [11].

Despite that, genome-wide association studies (GWAS) reported several genetic variants for COPD, none was tested in a middle-eastern population [6]. Furthermore, the interplay between VitD and the genetic determinants has not been yet investigated. To do so, we selected the top 10 GWAS SNPs reported to be highly significant with COPD (https://www.ebi.ac.uk/gwas) [6,12,13] and tested their association in 631 Lebanese individuals with and without COPD considering that VitD levels might also have a role. We also performed a meta-analysis combining our participants with different COPD populations from European and non-European origins.

## 2. Materials and Methods 

### 2.1. Ethics Statement

All the participants gave their written informed consent and were recruited following the latest version of the Declaration of Helsinki for Ethical Principles for Medical Research Involving Human Subjects. The institutional review board committee of Beirut Arab University gave ethical approval: 2019H-0053-HS-R-0308. 

### 2.2. Demographic Clinical and Biological Data Collection

COPD status was diagnosed using post-bronchodilator spirometry and, according to a post-bronchodilator forced expiratory volume in one second (FEV_1_) < 80% of the predicted value or a FEV_1_/forced vital capacity (FVC) less or equal to 70%. The demographic and clinical data were collected using a questionnaire. A body mass index (BMI) value of < 25 kg/m^2^ indicated a normal weight. Serum levels of total 25-hydroxyvitamin D (25(OH) D) were measured using Elecsys™ VitD total assay (Roche Diagnostics, Basel, Switzerland) and a calibrator. Blood samples were collected in EDTA tubes and genomic DNA was extracted from peripheral blood samples using QIAamp DNA Blood Mini Kit, Qiagen.

### 2.3. Genotyping Assays

The genotyping of ten SNPs’ was performed at the LGC group (Hoddesdon, United Kingdom) using KASP™ (Kompetitive allele specific PCR) genotyping assay. It is a FRET-based assay enabling accurate bi-allelic discrimination of known genetic variations. 

### 2.4. Statistical Analysis

Descriptive statistics were conducted and stated as frequencies and percentages for categorical variables and as means (±) standard deviation for continuous ones. 

A chi-squared test was performed to ascertain if the genotypes were in Hardy–Weinberg equilibrium. Normality was assessed using the Kolmogorov-Simonov test. Baseline comparisons between the two studied groups (Presence/Absence of COPD) were made using the Mann-Whitney U test for continuous variables. The level of significance was set at *p* ≤ 0.05.

A multivariate logistic regression model under the assumption of an additive model was employed to study COPD’s genetic associations. This model was adjusted for all potential confounding factors, including age, gender, BMI, VitD levels, and smoking. The interaction between rs6837671A>G x VitD levels was also evaluated within our model. The level of significance was set at *p* ≤ 0.01.

The statistical analyses, data management, and cleaning were executed using the SPSS (IBM Corp., Released 2013, SPSS Statistics for Windows Version 22.0, Armonk, NY, USA).

### 2.5. Meta-analysis with Chronic Obstructive Pulmonary Disease 

Meta-analysis was performed using Comprehensive Meta-Analysis software “V3”; calculations were performed using a fixed-effects method. Statistical heterogeneity among studies was assessed using Cochran’s Q, and the inconsistency $I^{2}$ tests, in which values above 25% and 50% were considered indicative of moderate and high heterogeneity, respectively [14]. The level of significance was set at *p* ≤ 0.05.

## 3. Results

The characteristics of the study participants were presented in Table 1. The total number of individuals was 631, with an average age of 46.8 years old. Whereas around 28% of the participants were diagnosed with COPD, around 60% were females and having low VitD levels. Body mass index did not differ between participants, as half of the individuals were obese. 57.5% were non-smokers, 5.2% were past smokers, and 37.1% were current smokers.

**Figure 1:**
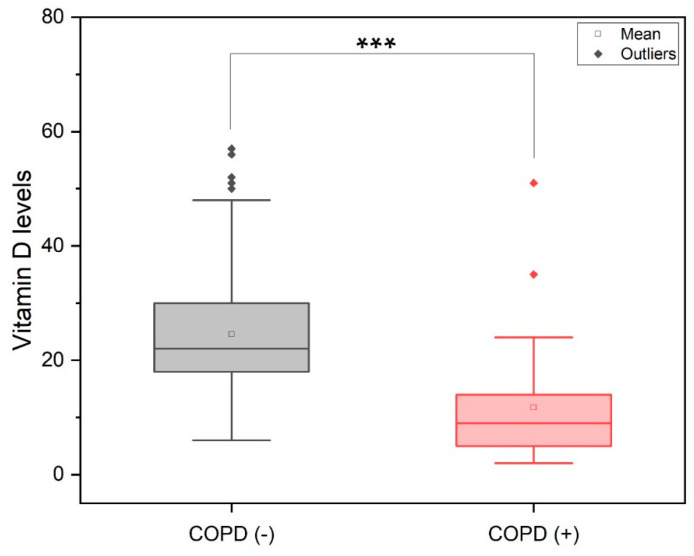
Vitamin D levels according to the status of chronic obstructive pulmonary disease. ^***^ Mann-Whitney test (*p* < 0.001).

When stratified according to VitD levels, the individuals with COPD had twice lower levels of VitD when compared to health ones (*p* < 0.001, Figure 1). Specifically, the VitD levels were < 30 ng/mL vs. ≥ 30 ng/mL, respectively, in individuals with and without COPD.

Two SNPs (rs113897301 in ADAM19 and rs7186831 in AC009163.5) showed no variation (monomorphic) in the in-house panel of samples and thus were excluded from the subsequent analysis (Supplementary Table S1). The remaining ones were in agreement with the Hardy–Weinberg equilibrium. In addition, While A is the minor allele of rs7733088 in the current study, G was shown to be the mean minor allele (calculated as mean because populations from different ancestry were included) in Hobbs et al. [6] (Supplementary Table S1). All the variables with a significant univariate result were selected for the multivariate analysis. A multivariate logistic regression model corrected for different confounding factors was used, procure that rs6837671 in *FAM13A* (Family with sequence similarity 13, member A) was associated with COPD (*p* = 0.01, Table 2). Among other dependent variables, age, BMI, low level of VitD, and smoking were found to increase COPD’s risk (*p* < 0.001, *p* < 0.001, and *p* = 0.013, respectively).

An interaction between rs6837671A>G and VitD levels, positively influencing the risk of COPD, was also observed (*p* < 0.001, Table 3). When taken together, the interaction between rs6837671 and low VitD levels increased the risk of being affected with COPD by 3.35 times. 

The stratification according to VitD status and the rs6837671 showed that the A allele was more prevalent in healthy individuals than in COPD, especially among individuals with low VitD levels (*p* = 0.039, Table 4). This result implies that the G allele is high in individuals with COPD, especially if they also have low VitD levels. 

**Figure 2:**
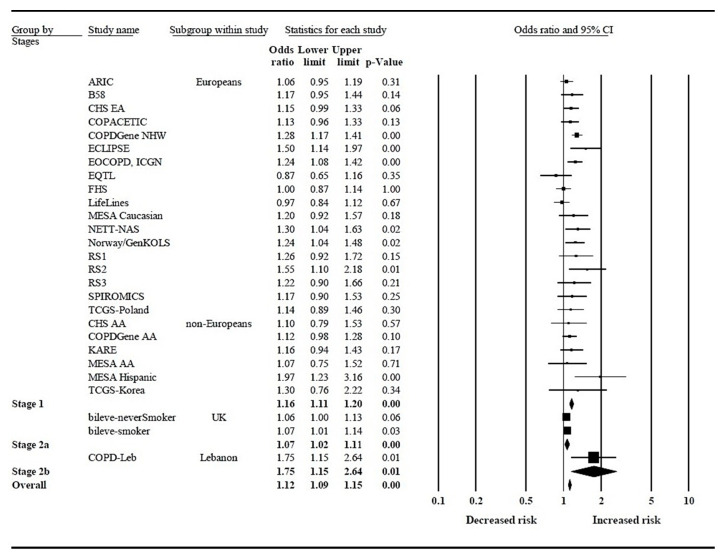
Forest plot for the association of rs6837671 in *FAM13A* and chronic obstructive pulmonary disease. Stage 1: discovery populations, stage 2a: replication in European populations, stage 2b: replication in non-European populations. Heterogeneity: Tau^2^ = 0.005, Chi^2^ = 48.720, df = 26 (*p* = 0.004); I^2^ = 46.6%. Test for overall effect: Z = 7.780 (*p* < 0.001).

A forest plot for meta-analysis of rs6837671A>G in *FAM13A* is shown in Figure 2. We included our study (named: COPD-Leb) in addition to 27 additional ones. We used a staged study design and examined overall meta-analysis P-values to determine the association of rs6837671 with COPD. The results showed that rs6837671G significantly increased the risk of COPD (OR = 1.12 and *p* < 0.001, Figure 2). The heterogeneity test showed a moderate heterogeneity between the different studies with *p* = 0.004 and I^2^ = 46.6%.

## 4. Discussion

We found that rs6837671A>G in *FAM13A* and VitD levels were associated with increased risk of COPD. When combined together, rs6837671A>G and VitD interacted in order to increase the risk of COPD. The stratification according to VitD status and the A allele of rs6837671 in *FAM13A* was more prevalent in healthy individuals than in COPD, and this difference was significant in individuals with low VitD levels. Finally, we have performed a meta-analysis by adding our study to the previously published COPD populations and found that the association between rs6837671A>G and COPD is now successfully replicated in Middle-Eastern individuals.

The role of VitD in pulmonary cell biology is complex and involves many processes, such as inflammation [8]. Despite no direct mechanistic implication, several epidemiological and experimental evidence highlights this connection’s relevance [8]. The relationship between VitD levels and COPD has always been an interest for the scientific community. While Black et al. found a strong association between serum levels of VitD and lung function (FEV1 and FVC) after adjustment for potential confounders in 14,091 adults in the USA [15], Forli et al. found that VitD deficiency (< 20 ng/mL) in more than the half of the individuals waiting for lung transplantation [16].

*FAM13A* is found on the long arm of chromosome 4 (4q22.1). It encodes a signal transduction protein expressed in the airway and alveolar type II epithelial cells and in lung macrophages of humans and mice organisms [17]. Although very little is known about *FAM13A* function [18], the transcriptomic analyses in various cell lines have demonstrated a consistent increase in its levels in response to hypoxia [18]. Therefore, *FAM13A* was considered as one of many “epithelial cell hypoxia genes” [18]. In non-small lung cell cancer, *FAM13A* was reported to be involved in tumor proliferation downstream of HIF (Hypoxia Inducible Factor)-1α and TGF-β [19]. *FAM13A* is also associated with idiopathic pulmonary disease, but its expression in lung tissues is similar between cases and controls and when stratified according to the genotype [20]. 

On the other hand, dietary-induced VitD deficiency was reported to magnify lung injury, alveolar inflammation, and hypoxia in a murine model showing intra-tracheal lipopolysaccharide [21]. Hypoxia harms the endothelium-dependent pulmonary artery relaxation in severe COPD cases [22]. Alveolar hypoxia and consequent hypoxemia are positively correlated with the severity of COPD [23]. Chronic hypoxemia predisposes the body to COPD’s adverse complications, including pulmonary hypertension and systemic inflammation [23]. Therefore, we concluded that rs6837671A>G in *FAM13A* and VitD might be associated with an increased risk of COPD through hypoxia in the lungs and specifically at the alveoli site. 

The emergence of high throughput genotyping and sequencing platforms (GWAS and next-generation sequencing) has allowed substantial progress in understanding the underlying genes influencing COPD risk. In this context, the largest multi-stage GWAS study conducted initially on 15,256 cases and 47,936 controls, with replication in 9,498 cases and 9,748 controls identified 22 loci, including 13 were novel [6]. Interestingly, nine of these thirteen variations were also associated with lung function in samples from the general population [6]. 

The study’s main limitation is that we did not have access to the spirometer results of a significant number of COPD patients (despite that they were used for diagnosis). This issue limited our capacity to test the association between rs6837671, VitD, and spirometric lung function tests such as FEV1 and FEV1/FVC. Therefore, we could not conclude if the association with COPD susceptibility could pass by these phenotypes (FEV1 and FEV1/FVC).

## 5. Conclusions

Our result that rs6837671 in *FAM13A* is associated with COPD replicates the previous findings of the largest GWAS study (60,000 subjects) in a Middle Eastern population. This SNP can now be considered a trans-ethnic risk factor for COPD since it showed significant signals in COPD cases from different ethnicities; Europeans, Asians, and now middle eastern. Future transcriptomic studies in whole blood of COPD patients and controls might reveal novel insights on the underlying pathological mechanisms.

## Figures and Tables

**Table 1:** Characteristics of the study participants.

	Participants (*n* = 631)
COPD status *n*(%)	172 (27.5)
Age (years)	46.75 ± 17.07
Gender (*n* = 631)	
Female n(%)	375 (59.4)
Male n(%)	256 (40.6)
BMI (kg/m^2^) (*n* = 625)	
Normal weight n(%)	311 (49.8)
Obesity n(%)	314 (50.2)
Vitamin D (*n* = 489)	
Normal n(%)	124 (19.7)
Low n(%)	365 (57.8)
Smoking (*n* = 630)	
Non-smoker	396 (62.8)
Smoker	234 (37.1)
MAF	
rs17486278A>C in *CHRNA5*	0.41
rs7733088G>A in *HTR4*	0.42
rs9399401T>C in *ADGRG6*	0.26
rs1441358T>G in *THSD4*	0.41
rs6837671A>G in *FAM13A*	0.25
rs11727735A>G *INTS12*-*GSTCD*	0.08
rs2047409C>T in *TET2*	0.45
rs2955083A>T in *EEFSEC*	0.12

Values are arithmetic mean ± SD for continuous variables. Categorical variables were shown as number (*n*) and percentages. COPD: chronic obstructive pulmonary disease. *n*: sample size. BMI: body mass index. MAF: minor allele frequency.

**Table 2:** Multiple logistic regression analysis with chronic obstructive pulmonary disease.

		COPD	
	OR	CI (95%)	*p*
Age	1.12	1.10–1.15	< 0.001
Gender			
Female	1	-	-
Male	1.02	0.61–1.70	0.946
BMI			
Normal weight	1	-	-
Obese	2.66	1.56–4.55	< 0.001
Vitamin D levels			
Normal	1	-	-
Low	3.10	1.82–5.27	< 0.001
Smoking			
Non-smoker	1	-	-
Smoker	2.64	1.23–5.69	0.013
rs17486278A>C in *CHRNA5*			
AA	1	-	-
CA	0.84	0.48–1.47	0.553
CC	0.63	0.29–1.37	0.241
rs7733088G>A in *HTR4*			
GG	1	-	-
GA	1.16	0.67–2.01	0.604
AA	1.22	0.59–2.50	0.590
rs9399401T>C in *ADGRG6*			
TT	1	-	-
TC	1.69	1.01–2.84	0.048
CC	2.27	0.91–5.65	0.079
rs1441358T>G in *THSD4*			
TT	1	-	-
TG	1.25	0.73–2.17	0.417
GG	1.04	0.49–2.23	0.911
rs6837671A>G in *FAM13A*			
AA	1	-	-
GA	1.53	0.90–2.59	0.117
GG	3.99	1.39–11.47	0.010
rs11727735A>G *INTS12*-*GSTCD*			
AA	1	-	-
GA	0.49	0.23–1.06	0.071
GG	0.59	0.04–8.35	0.697
rs2047409C>T in *TET2*			
CC	1	-	-
TC	0.76	0.44–1.32	0.326
TT	0.51	0.24–1.08	0.079
rs2955083A>T in *EEFSEC*			
AA	1	-	-
TA	0.47	0.25–0.91	0.025
TT	6.25	0.93–41.81	0.059

OR: odds ratio, CI: confidence interval, BMI: body mass index.

**Table 3:** Interaction analysis with chronic obstructive pulmonary disease.

		COPD	
	OR	CI (95%)	*p*
Age	1.15	1.09–1.21	< 0.001
Gender			
Female	1		
Male	0.63	0.21–1.91	0.413
BMI			
Normal weight	1	-	-
Obese	2.04	0.69–6.02	0.197
Smoking			
Non-smoker	1	-	-
Smoker	2.77	2.06–3.71	< 0.001
rs6837671A>G, *FAM13A* x Vitamin D levels	3.35	1.73–6.49	< 0.001

COPD: chronic obstructive pulmonary disease, OR: odds ratio, CI: confidence interval, BMI: body mass index.

**Table 4:** Frequency of A allele according to vitamin D and chronic obstructive pulmonary disease statuses.

	Low Vitamin D				Normal Vitamin D		
	*COPD* (-)	*COPD* (+)	*p*		*COPD* (-)	*COPD* (+)	*p*
Frequency A Allele	0.56	0.43	0.039		0.68	0.31	0.264

COPD: chronic obstructive pulmonary disease.

## Supplementary Materials

The following are available online at https://www.mdpi.com/2075-4426/11/2/84/s1, Table S1: Comparison of the allele frequencies between the current study with hobbs et al.

## Abbreviations

COPD	Chronic obstructive pulmonary disease
FAM13A	Family with sequence similarity 13, member A
FEV1	Forced expiratory volume in one second
FVC	Forced vital capacity
GWAS	Genome-wide association studies
VitD	Vitamin D
