# Supplementary material for: rs6837671A>G in FAM13A Is a Trans-Ethnic Genetic Variant Interacting with Vitamin D Levels to Affect Chronic Obstructive Pulmonary Disease
Source: J Pers Med. 2021 Jan 30;11(2):84. doi: 10.3390/jpm11020084 (PMC7912529; doi:10.3390/jpm11020084)
Supplement: Supplementary file 1 [file jpm-11-00084-s001.pdf]

Supplementary Table: Comparison of the allele frequencies between the current study with hobbs et al.

	COPD-Leb	Hobs et al		
SNP	Minor Allele Frequency	Risk Allele	Mean Allele Frequency	Range
rs17486278C in *CHRNA5*	0.41	C	0.35	0.24–0.44
rs7733088A in *HTR4*	0.42	A	0.6	0.47–0.69
rs9399401C in *ADGRG6*	0.26	T	0.72	0.61–0.75
rs1441358G in *THSD4*	0.41	G	0.33	0.19–0.55
rs6837671G in *FAM13A*	0.25	G	0.41	0.36–0.58
rs11727735G *INTS12-GSTCD*	0.08	A	0.94	0.93–0.99
rs2047409T in *TET2*	0.45	A	0.62	0.22–0.65
rs2955083T in *EEFSEC*	0.12	A	0.88	0.85–0.89
rs113897301 in *ADAM19*	0	AT	0.17	0.05–0.19
rs7186831 in *AC009163.5*	0	A	0.43	0.23-0.47

SNP: Single nucleotide polymorphisms.
